# Active Flavonoids from *Colubrina greggii* var. *greggii* S. Watson against Clinical Isolates of *Candida* spp.

**DOI:** 10.3390/molecules26195760

**Published:** 2021-09-23

**Authors:** Elda M. Melchor-Martínez, Juan F. Tamez-Fernández, Gloria María González-González, David A. Silva-Mares, Noemí Waksman-Minsky, Luis Alejandro Pérez-López, Verónica M. Rivas-Galindo

**Affiliations:** 1 Departamento de Química Analítica, Facultad de Medicina, Universidad Autonoma de Nuevo Leon, Av. Madero s/n, Colonia Mitras Centro, Monterrey 64460, Nuevo León, Mexico; elda.melchor@tec.mx (E.M.M.-M.); juan.tamezfrn@uanl.edu.mx (J.F.T.-F.); david.silvamr@uanl.edu.mx (D.A.S.-M.); noemi.waksmanmn@uanl.edu.mx (N.W.-M.); luis.perezlp@uanl.edu.mx (L.A.P.-L.); 2 School of Engineering and Sciences, Tecnologico de Monterrey, Monterrey 64849, Nuevo León, Mexico; 3 Departamento de Microbiología, Facultad de Medicina, Universidad Autonoma de Nuevo Leon, Av. Madero s/n, Colonia Mitras Centro, Monterrey 64460, Nuevo León, Mexico; gloria.gonzalezgn@uanl.edu.mx

**Keywords:** antifungal activity, *candida* spp., *colubrina greggii* var. *greggii*, bioassay-guided fractionation

## Abstract

*Candida* *albicans* is the most commonly implicated agent in invasive human fungal infections. The disease could be presented as minimal symptomatic candidemia or can be fulminant sepsis. Candidemia is associated with a high rate of mortality and high healthcare and hospitalization costs. The surveillance programs have reported the distribution of other *Candida* species reflecting the trends and antifungal susceptibilities. Previous studies have demonstrated that *C. glabrata* more frequently presents fluconazole-resistant strains. Extracts from Mexican plants have been reported with activity against pulmonary mycosis, among them *Colubrina greggii*. In the present study, extracts from the aerial parts (leaves, flowers, and fruits) of this plant were evaluated against clinical isolates of several species of *Candida* (*C. albicans*, *C. glabrata*, *C. parapsilosis*, *C. krusei*, and *C. tropicalis*) by the broth microdilution assay. Through bioassay-guided fractionation, three antifungal glycosylated flavonoids were isolated and characterized. The isolated compounds showed antifungal activity only against *C. glabrata* resistant to fluconazole, and were non-toxic toward brine shrimp lethality bioassay and in vitro Vero cell line assay. The ethyl acetate and butanol extracts, as well as the fractions containing the mixture of flavonoids, were more active against *Candida* spp.

## 1. Introduction

Over the past two decades there has been a dramatic increase in the incidence of systemic fungal infections related to immuno-compromised patients, cancer chemotherapy, or organ transplant recipients [1]. Although medical advances made it possible to lengthen the life of these patients, they are highly susceptible to fungal infections, the majority of them are contributing to an increase in the mortality and morbidity in healthy and immunocompromised patients [2]. In addition, antifungal drugs often exert multiple adverse effects and are occasionally dose-limiting. Although there seems to be a good number of antifungal drugs in clinical use, there are few options for therapeutic use [3]. Besides the toxicity produced for some drugs (polyenes, allylamines, azoles, and recently developed echinocandin class of molecules) [4], others are fungistatic and non-fungicides producing recurrence and other ones development cross-resistance (5-Flucytosine) [5]. The above mentioned represent a real problem due to prolonged treatments. New drugs like posaconazole, ravuconazole, micafungin, and anidulafungin are being researched and are promising [6].

*Candida* is the agent most frequently implicated in invasive fungal infections and now ranks as the fourth most common cause of nosocomial bloodstream infections (BSI). The overall increase in candidemia in recent years is complicated by the emergence of non-*C. albicans Candida* (NAC) species as both colonizers and pathogens causing fungal BSI. The extensive use of fluconazole could be a principal factor in the enhanced infections by NAC [7].

Studies reported in the USA reflected the following distribution of *Candida* spp.: *C. albicans* 46.8% and NAC 53.2% (*C. glabrata*, *C. parapsilosis*, *C. tropicalis*) [7]. In a similar study in Mexico, González et al. [8] found the following distribution: *C. albicans* 43.5% (10% were fluconazole resistant strains) and NAC 56.5% (*C. parapsilosis*, *C. glabrata*, *C. tropicalis*, and *C. krusei*) [8]. In the latter group, 10 to 25% were fluconazole-resistant strains. Fungal infection by *Candida* spp. is becoming a serious medical problem because of the difficulty of its control in immune-compromised individuals, and because of the emergence of multidrug-resistant fungi, it has emphasized the need to obtain new antifungal drugs that are more effective and secure drugs from plants with antifungal activity [9].

Our research group previously reported an in vitro screening of the antifungal activity of several plants from Northeast Mexico against some of the main etiological agents inducing pulmonary mycoses, *Candida albicans*, *Aspergillus fumigatus*, *Histoplasma capsulatum*, and *Coccidioides immitis* [10]. The hydroalcoholic extract from *Colubrina greggii* was active against *C. albicans* with a minimal inhibitory concentration of 125 μg/mL. The hexane, ethyl acetate, and butanol extracts from *C. greggii* showed activity against *C. albicans* ranging from 62 to 250 μg/mL. Later, the antifungal and antioxidant activities of the methanol extract of *C. greggi* were reported [11].

*Colubrina greggii* S. Watson var. *greggii* (Rhamnaceae) is a shrub or tree widely distributed in Mexico. Some common names are guajolote, guayal, guayul, manzanita, trampillo, trompillo, vara prieta [12]. Traditional use of this plant is for the treatment of abscess, liver sickness, asthma, tuberculosis, and ulcerations [13,14]. Several species of the *Colubrina* genus have been reported to contain: saponins, alkaloids, triterpenes, essential oils, and phenolic metabolites that may be responsible for the biological activity [15].

Here, we report three antifungal glycosylated flavonoids obtained from both ethyl acetate and butanol extracts from *Colubrina greggiii* var. *greggii* aerial parts, through bioassay-guided fractionation.

## 2. Results and Discussion

In a previous and non-published work by our research group, we studied the aerial parts from *Colubrina greggii* (Colubrina), *Salvia texana* (Salvia), *Euphorbia prostrata* (Golondrina), *Clematis drummondii* female (Barba de chivo hembra), *Clematis drummondii* male (Barba de chivo macho), and the roots of *Jatropha dioica* (Sangre de Drago) collected at “ejido El potrero”, Villaldama, Nuevo León, México. The six plants were evaluated against two strains (clinical isolates) from *C. albicans, C. parapsilosis, C. glabrata, C. tropicalis,* and *C. krusei* by microdilution assay according to M27-A2 CLSI protocol [16]. Extracts of ethyl acetate from *E. prostrata* and *C. greggii*, and butanol from *C. greggii*, showed the highest activity against *Candida* spp. Toxic effects on *Artemia salina* have been demonstrated from *C. greggii* hexane extract. Based on the above results, the ethyl acetate and butanol extracts from *C. greggii* were selected for bioassay-directed fractionation to obtain antifungal compounds.

### 2.1. Extracts and Bioassay-Guided Fractionation against Candida *spp*.

Six fractions were obtained by gravitational column chromatography on silica gel (AE-F1 to F6). Each fraction obtained was evaluated by antifungal assay against *C. albicans, C. parapsilosis, C. glabrata, C. tropicalis* y *C. krusei* by microdilution assay according to M27-A2 CLSI protocol. Fraction 3 (AE-F3) showed activity against *C. parapsilosis* (MIC = 125 μg/mL), *C. glabrata* (MIC = 16 μg/mL) and *C. albicans* (MIC = 500 μg/mL) (Table 1). Column chromatography on silica gel of the AE-F3 fraction yielded compound **1** in a low yield. The compound **1** showed inhibition growth of *C. glabrata* (MIC = 16 μg/mL), *C. parapsilosis* (MIC = 125 μg/mL), *C. albicans*, and *C. krusei* (MIC = 500 μg/mL). Fraction AE-F6 was fractionated by low-pressure chromatography Lobar column RP-18, and 8 mg of compound **2** were obtained, which was active against *C. glabrata* (MIC = 63 μg/mL).

**Figure 1:**
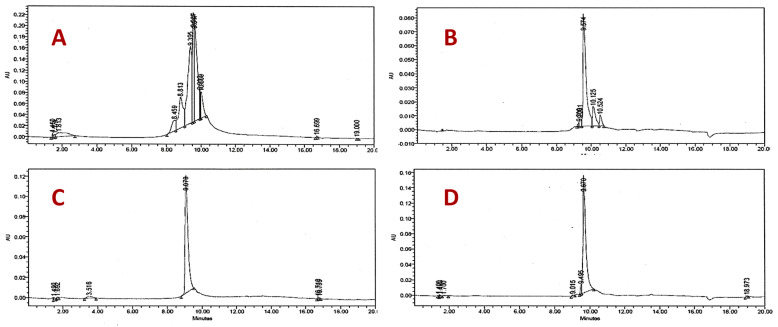
HPLC chromatograms of: (**A**) ethyl acetate extract; (**B**) compound **1** (t_R_ = 9.574 min); (**C**) compound **2** (t_R_ = 9.078 min); and (**D**) compound **3** (t_R_ = 9.670 min).

The butanol extract was fractionated by HPLC preparative equipment using an RP-18 column and four fractions were obtained (But-F1, But-F2, But-F3, But-F4) and evaluated against *Candida* spp in a similar way to the ethyl acetate extract. Fraction 4 (But-F4) demonstrated the highest activity against *C. parapsilosis* (MIC = 63 μg/mL), *C. glabrata* (MIC = 2 μg/mL), *C. krusei* (MIC = 32 μg/mL), *C. tropicalis* (MIC = 63 μg/mL), and *C. albicans* (MIC = 63 μg/mL) (Table 1). Fraction But-F4, was subjected to fractionation by Lobar RP-18 column and two compounds **2** and **3** were obtained in low yield. Compound **3** showed antifungal activity only against the resistant strains of *C. glabrata* with a MIC value of 16 μg/mL. The ethyl acetate extract and isolated compounds were analyzed by HPLC (Figure 1).

*C. glabrata* causes opportunistic infections in several parts of the human body. The microorganism has the ability to form biofilms and it is one of the basic virulence mechanisms, in addition to its production of hydrolytic enzymes such as proteinase, esterase, and phospholipase [17]. Adult patients with hematologic malignancies are more susceptible to its pathogenicity [18]. Additionally, in contrast to other *Candida* spp., *C.glabrata* lacks pseudohyphal form and expresses adhesin 1 (Epa1p) to enhance the attachment to infected host [19]. Compounds **1** and **3** showed the highest activity on 83 and 84 isolates of *C. glabrata (*azole-resistant strains) among the rest of the evaluated species. However, it is evident that both the ethyl acetate and butanol extracts, as well as the fractions containing the mixture of flavonoids, are more active against *Candida* spp. In addition, according to the NMR analysis of the active fractions, other flavonoids and epicatechins were identified, however, they were not obtained with adequate quantity and purity to perform a complete structural analysis and biological activity. Thus, they were not described in the present work.

Previous reports on the study of bioactive compounds from another variety of *Colubrina greggii* have been described. The organic crude extract of the root of *Colubrina greggii* var. *yucatanensis* were analyzed against *Bacillus subtilis*, *Staphyloccocus aureus,* and *Candida albicans*. A bioassay-guided led to the isolation and identification of chrysophanol as the compound responsible for the antimicrobial activity [15]. Extracts from seventeen plants including *Colubrina greggii* were tested against three Gram-negative bacterial strains (*Pseudomonas aeruginosa, Klebsiella pneumoniae,* and *Acinetobacter baumannii*), three Gram-positive bacterial strains (*Enterococcus faecalis* and two *Staphylococcus aureus strains*), and five species of yeasts (*Candida albicans, C. krusei, C. tropicalis, C. parapsilosis, and C. glabrata*). The hydromethanolic extracts from leaves and flowers of *Colubrina greggii* showed inhibitory activity against *E. fecalis* (MIC 250 µg/mL), *Candida glabrata* 84 (MIC 31.25 µg/mL), *C. albicans* 53 (MIC 125 µg/mL), *C. krusei* 168 (MIC 125 µg/mL), and *C. parapsilosis* 96 (MIC 62.5 µg/mL). At the present contribution has demonstrated higher inhibitory activity butanol and ethyl acetate extracts, from *C.greggii* on *C. glabrata* resistant strain compared with the activity of the hydromethanolic extracts reported by Salazar et al. [11].

### 2.2. Structural Identification of the Compounds and Their Antifungal Activity

**Figure 2:**
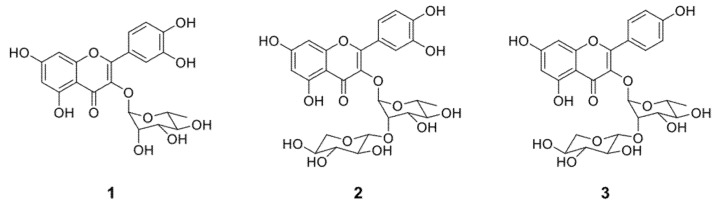
Structure of quercitrin (Compound **1**), quercetin 3-*O*-α-l-rhamnopyranosyl-l-(2→1)-*O*-β-xylopyranoside (Compound **2**) and kaempferol 3-*O*-α-l-rhamnopyranosyl-l-(2→1)-*O*-β-xylopyranoside (Compound **3**).

The structures of compounds **1**, **2,** and **3**, were established based on the ^1^H-NMR and ^13^C-NMR, LC-MS results, and for comparisons with published results (Figure 2, Supplementary Materials). The molecular formula of compound **1** was deduced by ESI-MS in negative mode showing a molecular ion peak [M − H]^−^ at *m*/*z* 447.0 corresponding to the formula C_21_H_20_O_11_. Structural analysis was made by ^1^H-NMR, ^13^C-NMR, and 2D-NMR. In agreement with all correlations found in the present work and the previous literature reports, its ^1^H-NMR characteristic signals 7.45 (d, *J* = 15.85 Hz), 7.32 (dd, *J* = 8.5 Hz), 6.80 (d*, J* = 8.5 Hz), 6.63 (s), and 6.35 (s) [20] made possible the identification as quercitrin (quercetin 3-*O*-α-l-rhamnoside).

Regarding the antifungal activity previously reported of compound **1**, MIC values below 250 μg/mL were obtained against *C. albicans* ATCC 18804, *C. tropicalis* ATCC 750, *C. krusei* ATCC 20298, and *C. parapsilosis* ATCC 20019 [21]. Antifungal activity of quercitrin and quercetin were evaluated on twelve clinical isolates of sensitive strains of yeast, *C. parapsilosis* (96), *C. tropicalis* (166), *C. krusei* (168), *C. albicans* (501, 498, 53, and ATCC 10231), and *C. glabrata* (507, 531, 587, 510, 493, 482). In that report, the isolates 510, 493, 482, 531 of *C. glabrata* were more sensitives to the treatment with quercitrin (MIC 7.8 µg/mL). The authors highlighted that the presence of the sugar moiety did not influence the antifungal activity against *Candida glabrata* [22].

Compound **2** showed a [M + H]^+^ ion at *m*/*z* 581.14 corresponding to the molecular formula of C_26_H_28_O_15_ by ESI-MS evaluation. A comparison was established based on a previous NMR analysis of the molecule [23], and 1D and 2D NMR signals of our samples to characterize as glycosylated flavonol composed for quercetin linked to rhamnose and xylose (quercetin 3-*O*-α-l-rhamnopyranosyl-l-(2→1)-*O*-β-xylopyranoside) (Figure 2).

This compound has been found in extracts from plants of the genus of *Zyzipus, Kalanchoe*, *Licania,* and *Miconia* [24,25,26]. For this compound, previous anti-*Candida* spp. activity has not been yet reported. Some studies have reported the inhibitory activity of the aglycone, quercetin on *C. albicans* with MIC values between 197 and 441 µg/mL [27].

Compound **3**, depicted a [M + H]^+^ ion at *m*/*z* 565.15 corresponding with the molecular formula of C_26_H_28_O_14_. The NMR characteristics signals on previous reports [23] were compared with the current NMR analysis to validate the identity of kaempferol 3-*O*-α-l-rhamnopyranosyl-l-(2→1)-*O*-β-xylopyranoside (Figure 1). Previous isolation from plants of the genus of *Moghania, Licania,* and *Ceanothus* has been reported [23,25,28,29]. The aglycone kaempferol showed inhibitory activity against six clinical isolates of *Candida glabrata* including one resistant to fluconazole with MIC values on 31.5 µg/mL [22]. Kaempferol-3-*O*-rutinoside, isolated from *Mitracarpus scaber* showed low antifungal activity on *Candida albicans* ATCC 14053 and ATCC10231 with MIC values of 500 µg/mL [30].

Considering the above information mentioned in the literature, there is a marked difference associated with the antifungal assay when it was carried out with *Candida* spp. obtained from the American Type Culture Collection (ATCC) or derived from clinical isolates. The relevance of the present study is the evaluation against species and strains that are persistent and resistant to fluconazole in patients from our region, Monterrey, Mexico. Finally, further studies in which aglycones and glycosides simultaneously analyzed are required, since this could determine the influence of the sugar moiety on antifungal activity against resistant strains of *Candida* spp.

### 2.3. Cytotoxicity Aassay

The safety of bioactive compounds from plants is a critical point, several assays have been established to provide information on their toxicity on normal systems. The proliferation of cell lines such as fibroblast is commonly measured [31]. The suitable methods should be able to detect the global toxicity of any extract, fraction, or compound. Also, they should offer advantages such as sensitivity, specificity, quickness, etc. The Artemia salina assay is commonly used, and described as economic, rapid, sensitive, accurate, and reliable to detect toxic compounds [32].

Studies about the medicinal potential of *Miconia albicans* described the characterization and toxicity effect of the fruit extracts from this plant. The authors reported higher content of phenolic compounds and flavonoids among them, quercetin, kaempferol, and their glycosilated derivates by UHPLC-QTOF-MS/MS. In favor of our results, their extracts presented approximately 95% viability after 24 h of exposure to 1000 μg/mL on in vitro assay carried out on the Vero cell line [33]. However, there are no previous reports that demonstrate the toxicity of compounds **1**, **2**, and **3** on Vero cell lines. The brine shrimp lethality test revealed that the isolated compounds were not toxic as their LC_50_ values (with 95% confidence interval) showed in Table 2, in agreement with Anderson et. al. that highlighted toxic compounds with values below 200 µg/mL [34]. The higher antifungal activity of the compounds **1**, **2**, and **3** on the clinical isolated *C. glabrata* resistant to fluconazole and their null toxicity contributes to the knowledge to propose them as candidates for further studies.

## 3. Materials and Methods

### 3.1. General Procedures

Commercially available solvents were used for the purification and analysis process (Fermont, Mexico and J.T. Baker^®^, New Jersey, USA). The 1D and 2D NMR experiments were performed on a Bruker AVANCE III HD 400 MHz (Bruker, Billerica, MA, USA). Chemical shifts are analyzed in ppm (δ) referenced to MeOD (Merck, Darmstadt, Germany) with δ 3.31 ppm for ^1^H and 49.00 for ^13^C. Mass Spectra (MS) data were acquired on a liquid chromatography-mass spectrometer (TOF) using electrospray ionization/APCI 6200 (Agilent, Santa Clara, CA, USA); and on a mass spectrometer with triple quadrupole Sciex API6500 Qtrap using electrospray ionization in negative mode (Sciex, Framingham, MA, USA). All fractions and purity of compounds were monitored by Thin-layer chromatography (TLC) on aluminum sheets, precoated with silica gel 60 F_254_ (Merck, Darmstadt, Germany), and HPLC-DAD using a Waters 2996 Analytical Liquid Chromatograph equipped with a Diode Array Detector (Waters^TM^, Milford, MA, USA) and a Chromolith Performance RP-18e (100–4.6 mm) column (Merck, Darmstadt, Germany). The analysis was carried out in a mixture of methanol/water (30:70, *v*/*v*) for fractions and (40:60, *v*/*v*) for the purified compounds on an isocratic mode. Ten microliters of each fraction were analyzed at a flow rate of 1 mL/min and detected at 254 nm. Column chromatography (CC) was carried out by 40–63 μm silica gel (Merck, Darmstadt, Germany) at low pressure. The structures of compounds **1**, **2**, and **3**, were established based on the ^1^H-NMR and ^13^C-NMR, LC-MS results, and comparisons with published results.

### 3.2. Collection of the Plant Material

Aerial parts including leaves, flowers, and fruits from *Colubrina greggii* var. *greggii* S. Watson were collected at “El Potrero”, Villaldama, Nuevo León, Mexico, in June 2005 and were authenticated at the Institutional Herbarium of the Facultad de Ciencias Biológicas, Universidad Autonoma de Nuevo Leon (UANL). A voucher specimen (No. UAN-12284) was deposited.

### 3.3. Preparation of Plant Extracts and Bioassay-Guided Fractionation

Aerial parts of the plant were dried at room temperature. The dried plant was ground into powder (200 g) and extracted with an ethanol:water (90:10) mixture and the obtained extracts were filtered and evaporated under low pressure at 40 °C (Büchi Labortechnik, Essen, Germany). The hydroalcoholic extracts were subjected to differential extraction with hexane, ethyl acetate, and butanol, and the obtained extracts were evaporated in a rotary evaporator. The ethyl acetate extract (200 mg) was fractionated by gravitational column chromatography on silica gel and eluted with an ethyl acetate-acetone gradient yielding six fractions (AE-F1 to AE-F6). Each fraction was evaluated for antifungal activity by microdilution assay and fractions (AE-F3 and AE-F6) showed higher activities. AE-F3 (50 mg) was purified by CC of silica gel with an ethyl acetate-acetone gradient; to led to compound **1**. AE-F6 (67 mg) was purified by column chromatography Lobar RP-18 (low pressure) with methanol-water 50:50 proportion and compound **2** was isolated. Each fraction obtained during separation procedures of ethyl acetate extract was analyzed by TLC (aluminum sheets, silica gel 60 F_254_) with ethyl acetate-water-formic acid-acetic-acid (25:2:1:1) and by means analytical HPLC in a Waters 2996 chromatograph equipped with a Chromolith performance column (RP-18e, 100 × 4.6 mm) and using an isocratic system (methanol-water 30:70).

The butanol extract (200 mg) was subjected to separation by HPLC preparative equipment using an RP-18 column and eluted with methanol-water 30:70 proportion. Four fractions were obtained and evaluated against *Candida* spp. Fraction 4 (But-F4) resulted in the most active, and 35 mg were obtained and fractionated by Lobar RP-18 column, with methanol-water gradient (50:50 to 90:10) and two compounds were isolated, compounds **2** and **3**. Each fraction obtained during separation procedures of butanol extract was analyzed by TLC with chloroform-acetic acid-methanol-water (13:8:3:3) and by means analytical HPLC in a Chromolith performance column (RP-18e, 100 × 4.6 mm) and using isocratic system (methanol-water 40:60). Optimization of the isolation procedure was performed to reach a good yield for the in vitro cell line evaluation. The hydroalcoholic extract was precipitated with acetone and the supernatant was dried in vacuo. The chlorophyll contents of the supernatant were eliminated using SPE C18 cartridges (Alltech^TM^, Sagle, ID, USA), and the samples were eluted with 8 mL of 50%, 70%, and 100% MeOH. The 50% MeOH fraction was evaporated *in vacuo* and subjected to FLASH column chromatography (silica gel 60 for column chromatography, 0.040–0.063 mm, Merck Millipore^®^, Darmstadt, Germany) using EtOAc-MeOH-Water-Acetic Acid (25:1:1:0.1) as eluent. Fractions containing the isolated compounds were evaporated and precipitated with CH_2_Cl_2_ to afford compounds **1** (14.6 mg), **2** (56.2 mg), and **3** (13.9 mg).

### 3.4. Candida spp. Isolates and Antifungal Activity Evaluation

Clinical isolates were provided by the Microbiology Department of the Medical School, UANL: isolates 97 and 98 of *C. albicans*, isolates 83 and 84 of *C. glabrata*, isolates 95 and 96 of *C. parapsilosis*, isolates 105 and 166 of *C. tropicalis,* and isolates 137 and 168 of *C. krusei*.

Inoculates from the above-mentioned isolates were prepared and growing on Sabouraud agar. The extracts were tested using microdilution assay according to the protocol M27-A2 defined by the Clinical Laboratory Standards Institute [34]. Two clinical isolates were used for each species of *Candida*, and each test was made in duplicate. Isolates 83 and 84 of *C. glabrata* are resistant strains to fluconazole. Serial two-fold dilutions ranging from 2 to 500 μg/mL were made for all extracts, fractions, and isolated compounds. The minimal inhibitory concentration (MIC) was defined as the lowest tested extract concentration that inhibits 100% of organism growth compared to the growth of the drug-free control.

### 3.5. Cytotoxicity Assay

#### 3.5.1. Brine Shrimp Lethality Bioassay

In order to evaluate the toxicity of the compounds with antifungal activity obtained during separation, a test of lethality to *Artemia salina* brine shrimp was made [35]. Concentrations of 1, 10, 100, and 500 ppm of each active fraction and each compound were tested. The number of dead larvae was recorded and used to calculate the Lethal Concentration Medium (LC_50_) and 95% confidence intervals were determined from the 24 h counts using the Finney Probits analysis software. LC_50_ values greater than 200 ppm were considered non-toxic [34].

#### 3.5.2. In Vitro Cytotoxic Assay on Vero Cell Line

The cytotoxicity assay was performed by the 3-(4,5-dimethylthiazol-2-yl) 2,5-diphenyltetrazolium bromide (MTT) assay as previously described [36]. The Vero cell line was maintained in DMEM supplemented with 2% FBS, streptomycin, and penicillin at 1%. All cell cultures were incubated at 37 °C in a humidified atmosphere of 5% CO_2_. Cells were harvested, counted, and transferred into 96 well plates and incubated for 24 h prior to the addition of test compounds. Serial dilutions of test samples were prepared by dissolving compounds in DMSO followed by dilution with DMEM to yield the final DMSO concentration in the assay well as 1%. Stock solutions of fractions and compounds **1**, **2**, and **3** were prepared at 1 mg/mL and diluted further to yield concentrations ranging from 2000–15.6 μg/mL. The plates were incubated for 48 h. MTT (3-[4,5-dimethylthiazol-2-yl]-2,5-diphenyltetrazolium bromide) (5 mg) was dissolved in 1 mL of Phosphate Buffer Solution (PBS) and 10 μL of it was added to each of the 96 wells. The plates were incubated at 37 °C for 3 h. The solution in each well-containing media, unbound MTT and dead cells were removed by suction and 100 μL of DMSO was added to each well. The plates were then shaken and optical density was recorded using a microplate reader at 540 nm. Doxorubicin was used as positive control and DMSO as solvent control. The CC_50_ was determined as the concentration of extract required reducing cell viability by 50%, taking as 100%, the untreated cells. The experiments were performed in triplicate for each extract. In parallel, we performed the same experiment using untreated cells as a control.

### 3.6. Statistical Analysis

The analysis for the biological activity was carried out in triplicate. The data were reported as means ± standard deviation meaning. The data from the toxicological test on *Artemia salina* were analyzed with PROBIT software to determine LC_50_.

## 4. Conclusions

Three glycosylated flavonoids were isolated from *Colubrina greggii* var. *greggii* aerial parts extracts, through bioassay-guided fractionation. These compounds were characterized as quercetin 3-*O*-α-l-rhamnoside (compound **1**, quercitrin), quercetin 3-*O*-α-L rhamnopyranosyl-l-(2→1)-*O*-β-xylopyranoside (compound **2**), and kaempferol 3-*O*-α-l-rhamnopyranosyl-l-(2→1)-*O*-β-xylopyranoside (compound **3**). The mono-glycosylated flavonoid (**1**) showed activity against various species of *Candida*, while the di-glycosylated flavonoids only showed activity against *C. glabrata* (fluconazole-resistant isolates). In fact, the ethyl acetate and butanol extracts, as well as the fractions containing the mixture of flavonoids, resulted more active against *Candida* spp. All compounds were non-toxic toward the lethality test with *Artemia salina* and in vitro Vero cell assay. This is the first research report about active isolated compounds from *Colubrina greggii* var. *greggii*, and that support the use of this plant in Mexican traditional medicine.

## Figures and Tables

**Table 1:** Antifungal activity of fractions and compounds obtained from the aerial parts of *C. greggii* var *greggii*.

Extracts, Fractions, and Compounds	MIC (µg/mL)									
	Cg	Cg	Cp	Cp	Ca	Ca	Ct	Ct	Ck	Ck
	*83*	*84*	*95*	*96*	*97*	*98*	*105*	*166*	*137*	*168*
Ethyl Acetate extract	16	16	32	32	32	125	63	63	16	16
Butanol extract	2	2	63	63	63	63	250	125	16	16
Hexane extract	63	63	125	125	250	>500	>500	>500	125	125
Fraction AE-F1	>500	>500	>500	>500	250	>500	>500	>500	>500	>500
Fraction AE-F2	>500	>500	>500	>500	>500	>500	>500	>500	>500	>500
Fraction AE-F3	16	16	125	125	500	500	>500	>500	>500	>500
Fraction AE-F4	>500	>500	>500	>500	>500	>500	>500	>500	>500	>500
Fraction AE-F5	>500	>500	>500	>500	>500	>500	>500	>500	>500	>500
Fraction AE-F6	63	63	>500	>500	>500	>500	>500	>500	>500	16
Fraction But-F1	2	>500	>500	64	>500	>500	>500	125	>500	64
Fraction But-F2	2	>500	>500	250	>500	>500	>500	>500	>500	>500
Fraction But-F3	4	>500	>500	>500	>500	>500	>500	>500	>500	>500
Fraction But-F4	2	2	63	63	63	63	63	63	32	32
Compound 1	16	16	125	125	500	500	>500	>500	500	500
Compound 2	63	63	>500	>500	>500	>500	>500	>500	>500	>500
Compound 3	16	16	>500	>500	>500	>500	>500	>500	>500	>500
Fluconazol	32	63	0.5	1	4	4	2	2	32	4

MIC: Minimal inhibitory Concentration. Fungi: Ca, *Candida albicans*; Cp, *C. parapsilosis*; Cg, *C. glabrata*; Ct, *C. tropicalis* and Ck, *C. krusei*.

**Table 2:** In vitro toxicity data of compounds **1**, **2**, and **3** on Vero cell line and *Artemia salina* assay.

Compounds	Vero Cell Line CC_50_ (µg/mL) ^a^	*Artemia Salina* LC_50_ (µg/mL) ^a^
1	1659.6 ± 81	>500
2	>2000	322.8 ± 0.009
3	>2000	254.8 ± 0.024

^a^ Results are expressed as a mean (n = 3) ± SD.

## Data Availability

Not applicable.

## Supplementary Materials

The following are available online, Figure S1: ^1^H NMR spectrum (Methanol-*d*_4_, 400 MHz) of compound **1**, Figure S2: ^13^C NMR spectrum (Methanol-*d*_4_, 100 MHz) of compound **1**, Figure S3: ^1^H NMR spectrum (Methanol-*d*_4_, 400 MHz) of compound **2**, Figure S4: ^13^C NMR spectrum (Methanol-*d*_4_, 100 MHz) of compound **2**, Figure S5: ^1^H NMR spectrum (Methanol-*d*_4_, 400 MHz) of compound **3**, Figure S6: ^13^C NMR spectrum (Methanol-*d*_4_, 100 MHz) of compound **3**, Figure S7: ESI- Mass spectrum of compound **1**, Figure S8: ESI- Mass spectrum of compound **2**, Figure S9: ESI- Mass spectrum of compound **3**.
