# Supplementary material for: Active Flavonoids from Colubrina greggii var. greggii S. Watson against Clinical Isolates of Candida spp
Source: Molecules. 2021 Sep 23;26(19):5760. doi: 10.3390/molecules26195760 (PMC8510013; doi:10.3390/molecules26195760)
Supplement: Supplementary file 1 [file molecules-26-05760-s001.zip › molecules-1340591-supplementary.pdf]

Supporting Information

Active Flavonoids from *Colubrina greggii* var. *greggii* S. Watson against Clinical Isolates of *Candida* spp.

Elda M. Melchor-Martínez [1,2], Juan F. Tamez-Fernández [1], Gloria González-González [3], David A. Silva-Mares [1], Noemí Waksman-Minsky [1], Luis Alejandro Pérez-López [1] and Verónica M. Rivas-Galindo [1,*]

[1] Departamento de Química Analítica, Facultad de Medicina, Universidad Autonoma de Nuevo Leon, Av. Madero s/n, Colonia Mitras Centro, Monterrey 64460, Nuevo León, México; elda.melchor@tec.mx (E.M.M.-M.); juan.tamezfrn@uanl.edu.mx (J.F.T.-F.); david.silvamr@uanl.edu.mx (D.A.S.-M.); noemi.waksmanmn@uanl.edu.mx (N.W.-M.); luis.perezlp@uanl.edu.mx (L.A.P.-L.); veronica.rivasgl@uanl.edu.mx (V.M.R.-G.)

[2] School of Engineering and Sciences, Tecnologico de Monterrey, Monterrey 64849, Nuevo León, Mexico

[3] Departamento de Microbiología, Facultad de Medicina, Universidad Autonoma de Nuevo Leon, Av. Madero s/n, Colonia Mitras Centro, Monterrey 64460, Nuevo León, México; gloria.gonzalezgn@uanl.edu.mx

* Correspondence: veronica.rivasgl@uanl.edu.mx; Phone: +(52)-818-329-4185

S. No.	Contents	Page No.
1	NMR spectra of compounds 1, 2, and 3.	S3-S8
2	Mass spectra of compounds 1, 2, and 3.	S9-S11

List of Figures	Page No.
**Figure S1.** ^{1}H NMR spectrum (Methanol-*d*4, 400 MHz) of compound **1**.	**S3**
**Figure S2.** ^{13}C NMR spectrum (Methanol-*d*4, 100 MHz) of compound **1**.	**S4**
**Figure S3.** ^{1}H NMR spectrum (Methanol-*d*4, 400 MHz) of compound **2**.	**S5**
**Figure S4.** ^{13}C NMR spectrum (Methanol-*d*4, 100 MHz) of compound **2**.	**S6**
**Figure S5.** ^{1}H NMR spectrum (Methanol-*d*4, 400 MHz) of compound **3**.	**S7**
**Figure S6.** ^{13}C NMR spectrum (Methanol-*d*4, 100 MHz) of compound **3.**	**S8**
**Figure S7** ESI-MS spectrum of compound 1	**S9**
**Figure S8** ESI-MS spectrum of compound 2	**S10**
**Figure S9** ESI-MS spectrum of compound 3	**S11**

1. NMR Spectra.

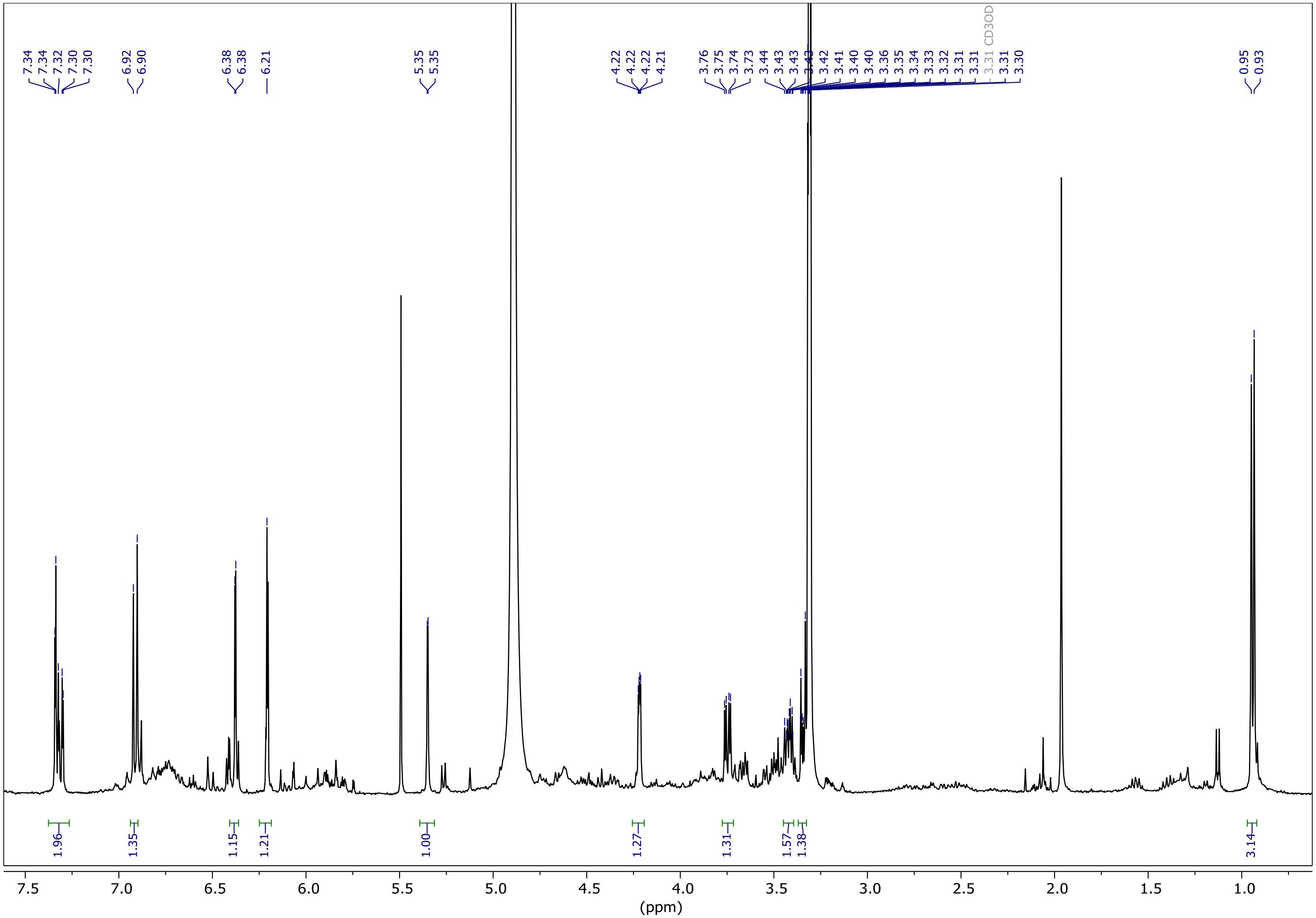

Figure S1. ^{1}H NMR spectrum (Methanol-*d*4, 400 MHz) of compound 1.

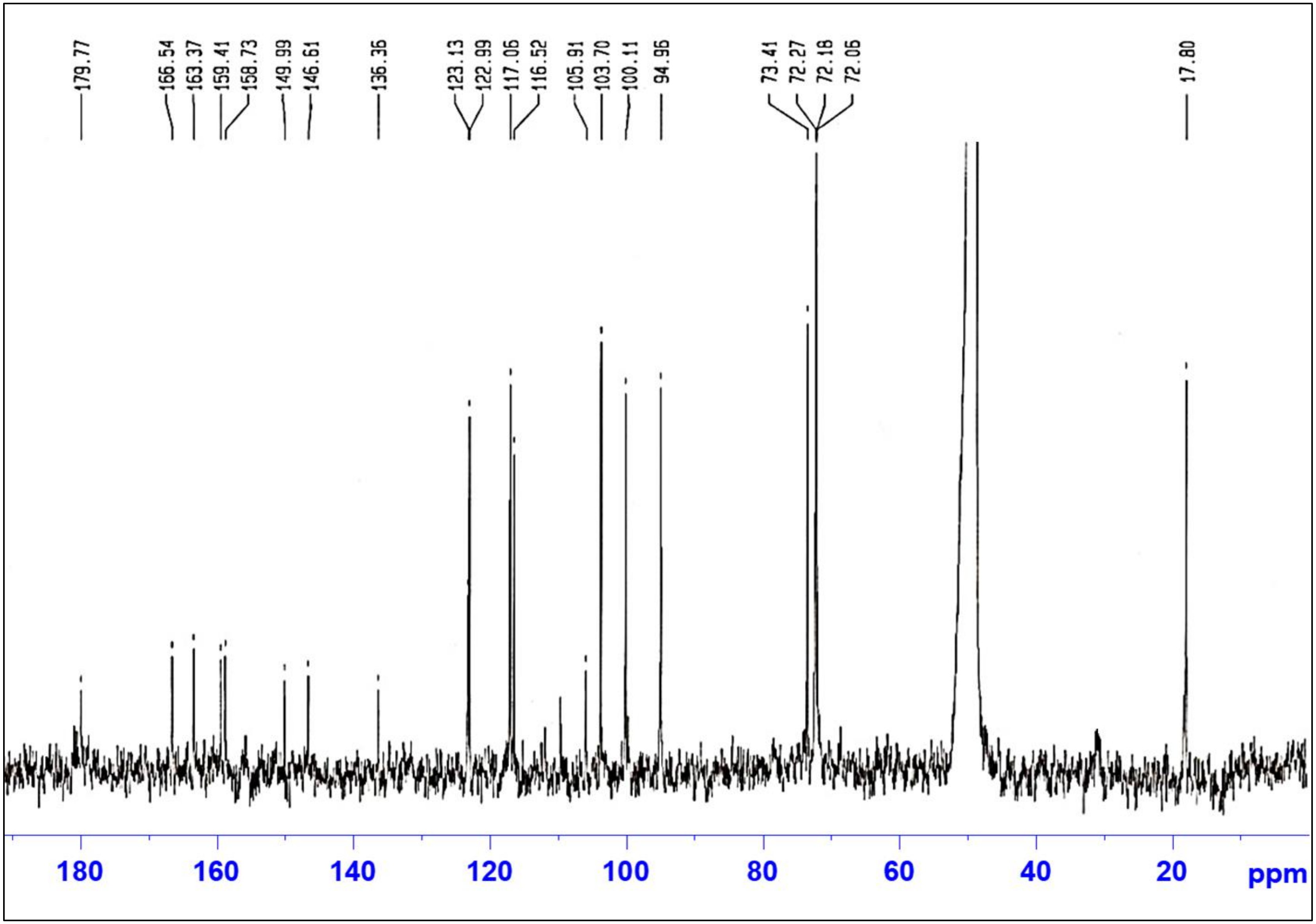

Figure S2. ^{13}C NMR spectrum (Methanol-*d*4, 100 MHz) of compound 1.

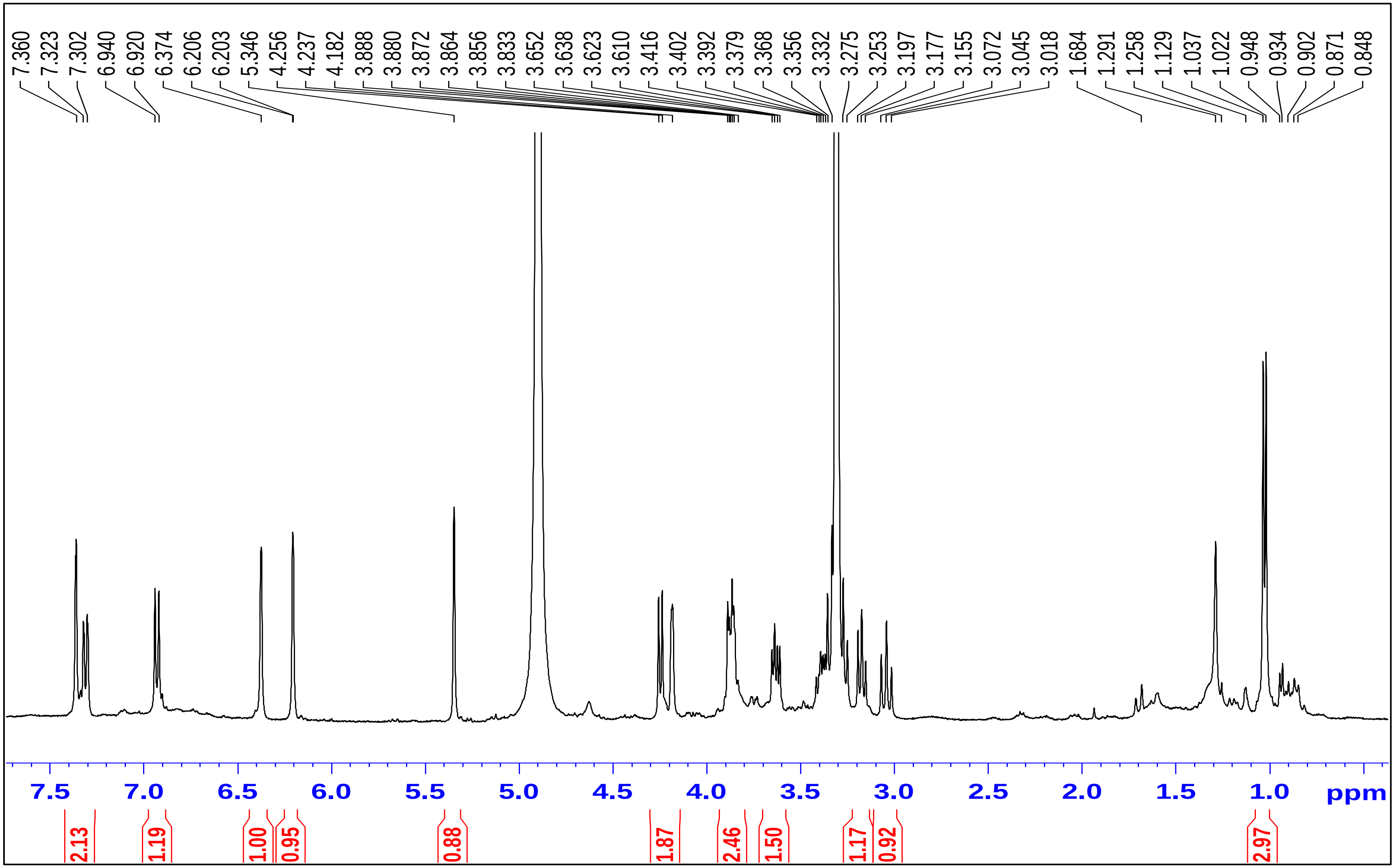

Figure S3. ^{1}H NMR spectrum (Methanol-*d*4, 400 MHz) of compound 2.

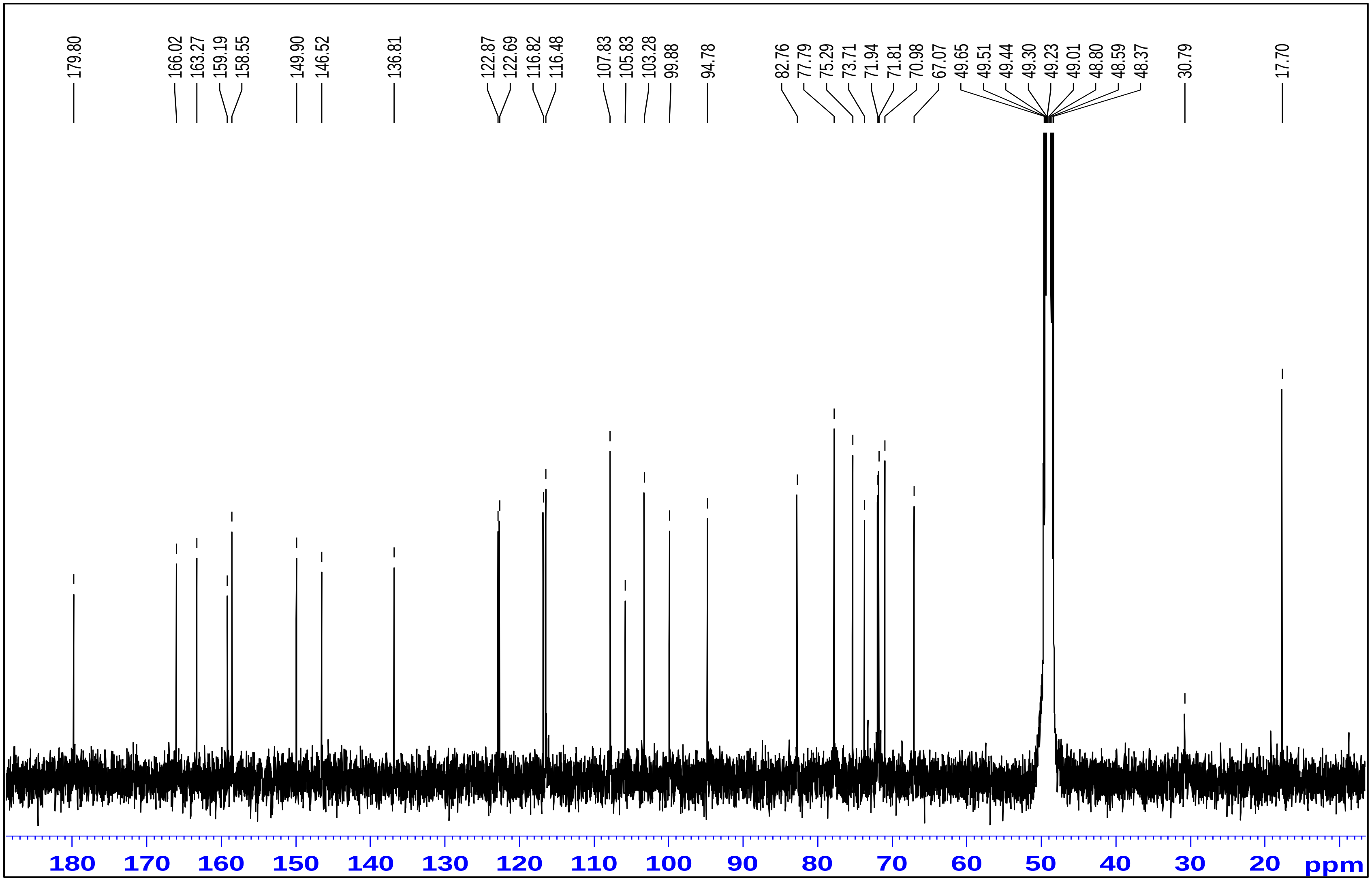

Figure S4. ^{13}C NMR spectrum (Methanol-*d*4, 100 MHz) of compound 2.

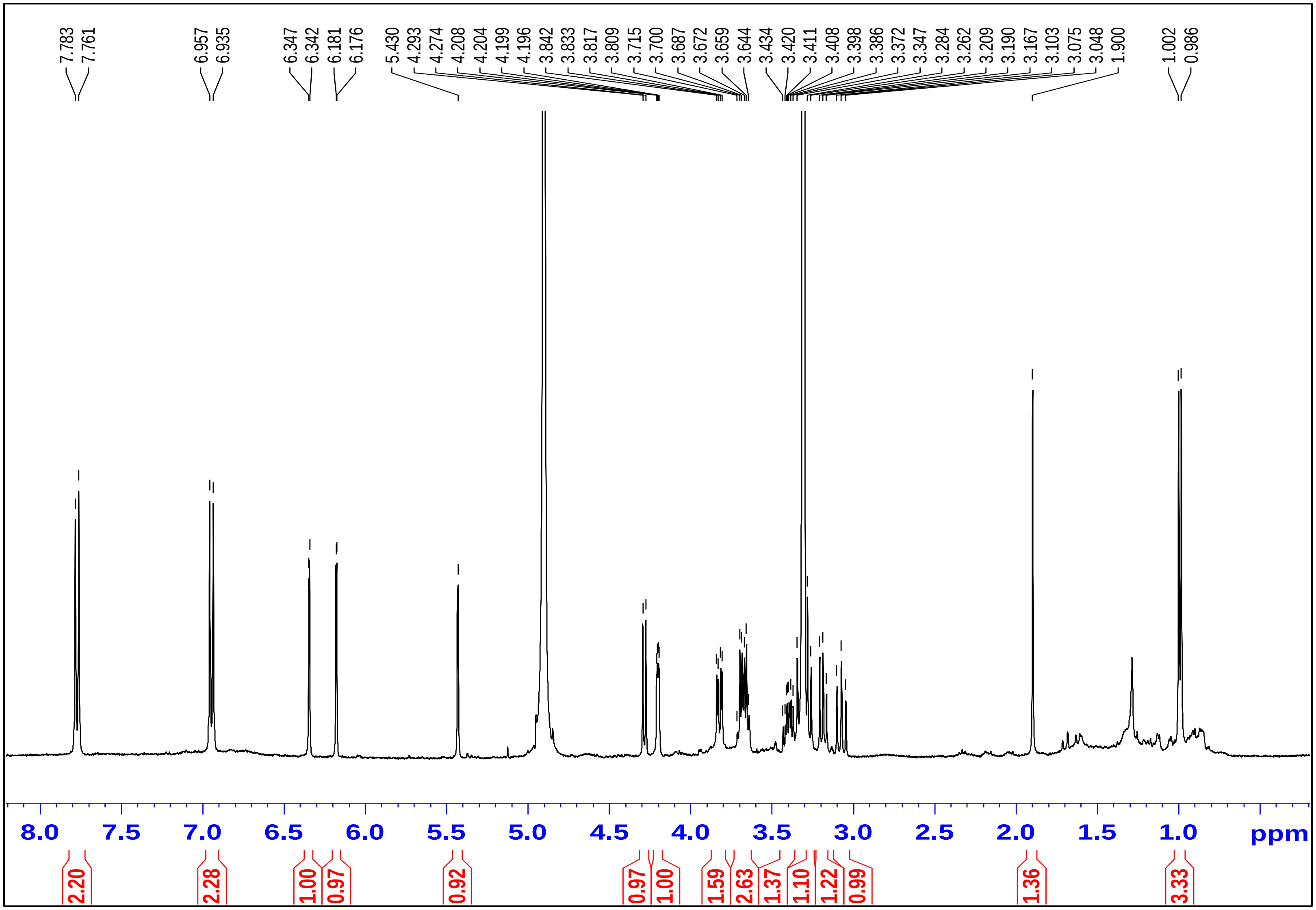

Figure S5. ^{1}H NMR spectrum (Methanol-*d*4, 400 MHz) of compound 3.

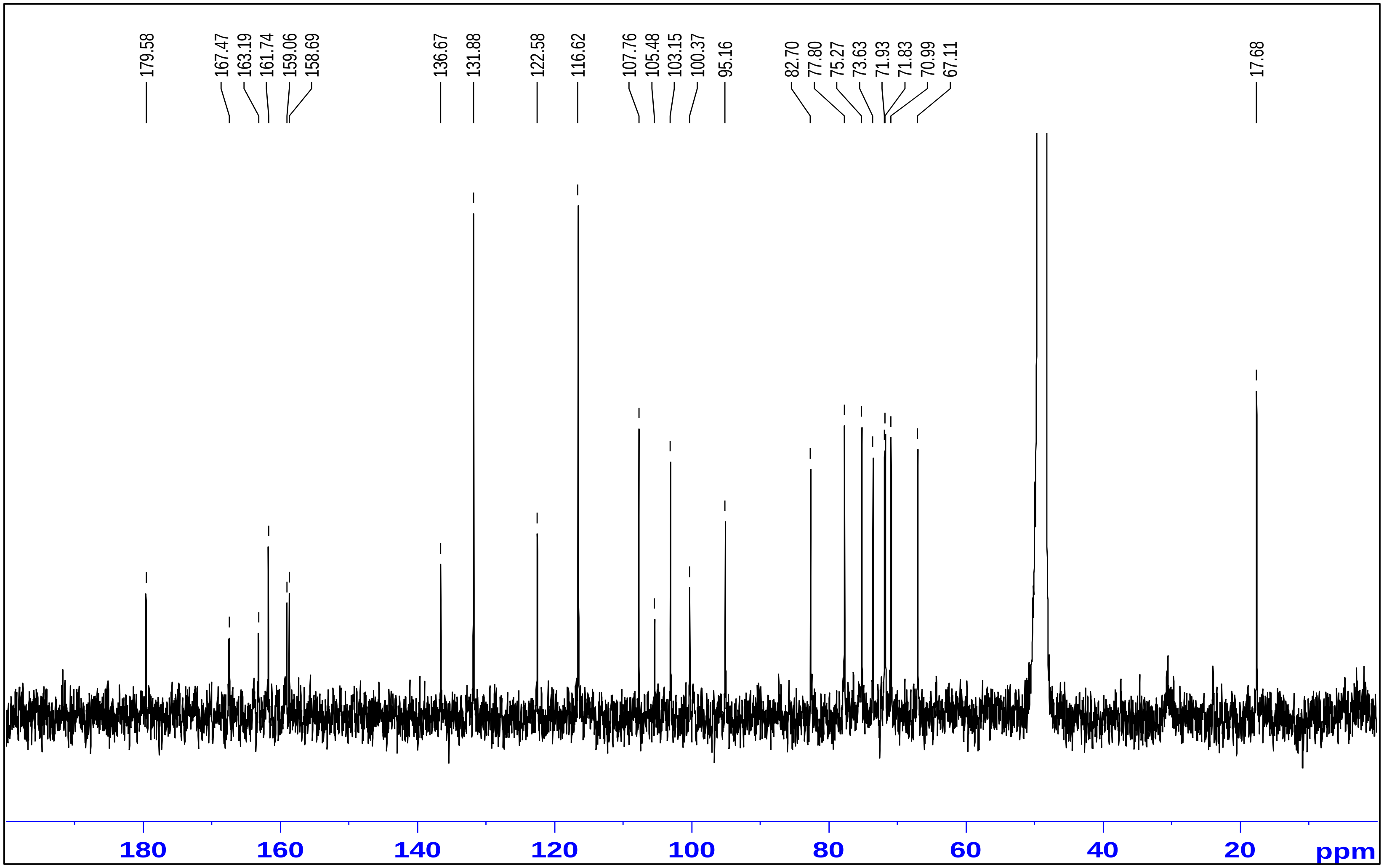

Figure S6. ^{13}C NMR spectrum (Methanol-*d*4, 100 MHz) of compound 3.

1. Mass Spectra.

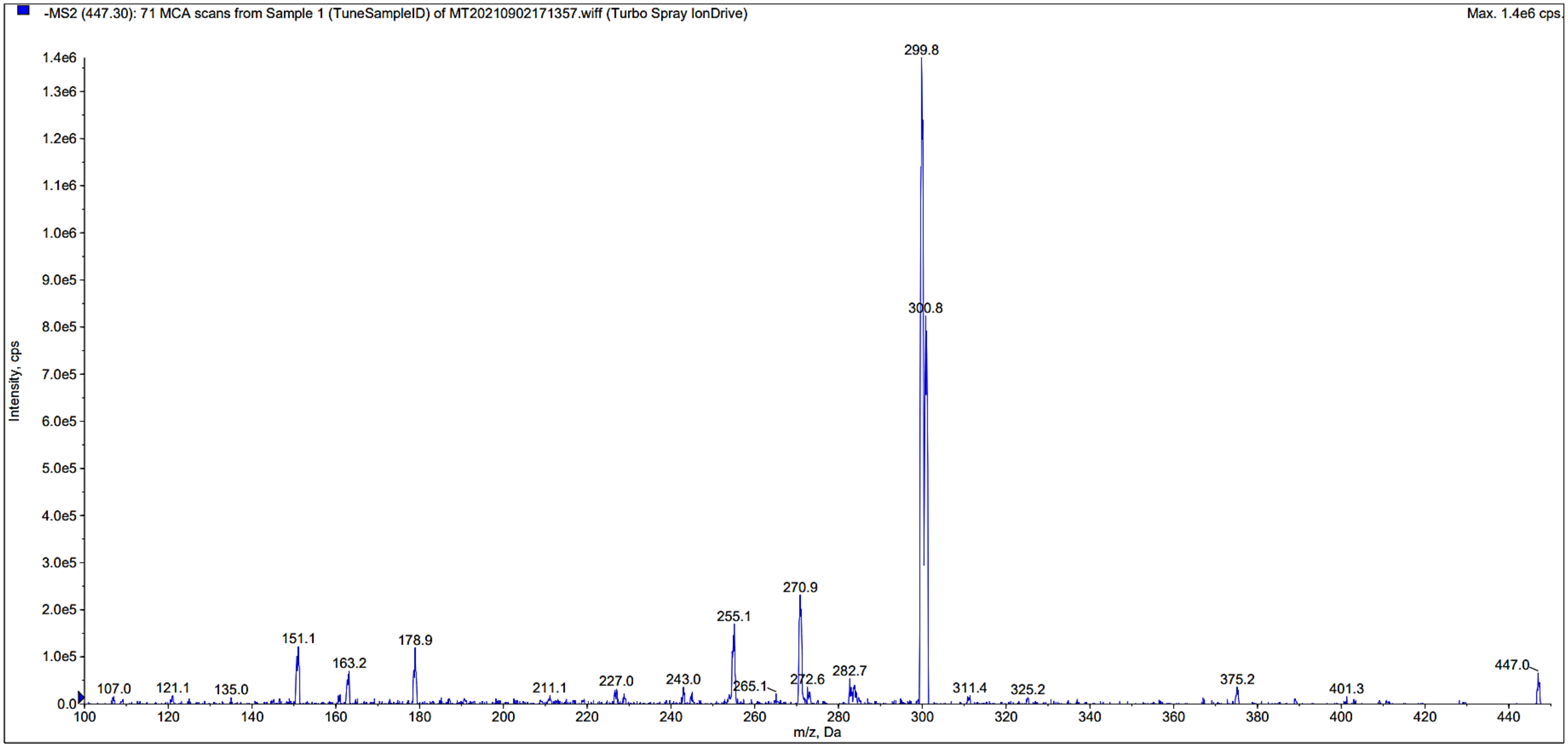

Figure S7 ESI-MS spectrum of compound 1

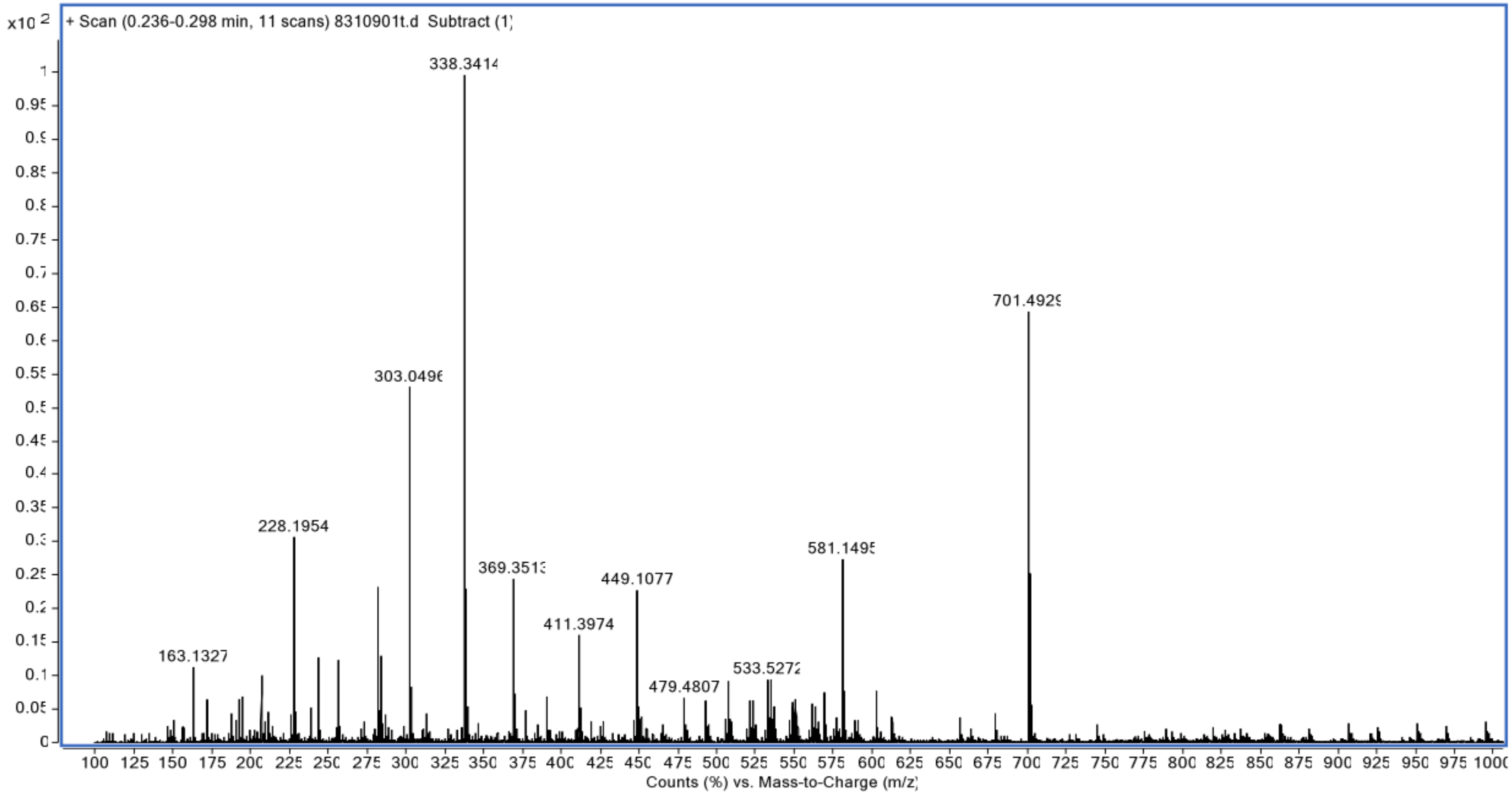

Figure S8 ESI-MS spectrum of compound 2

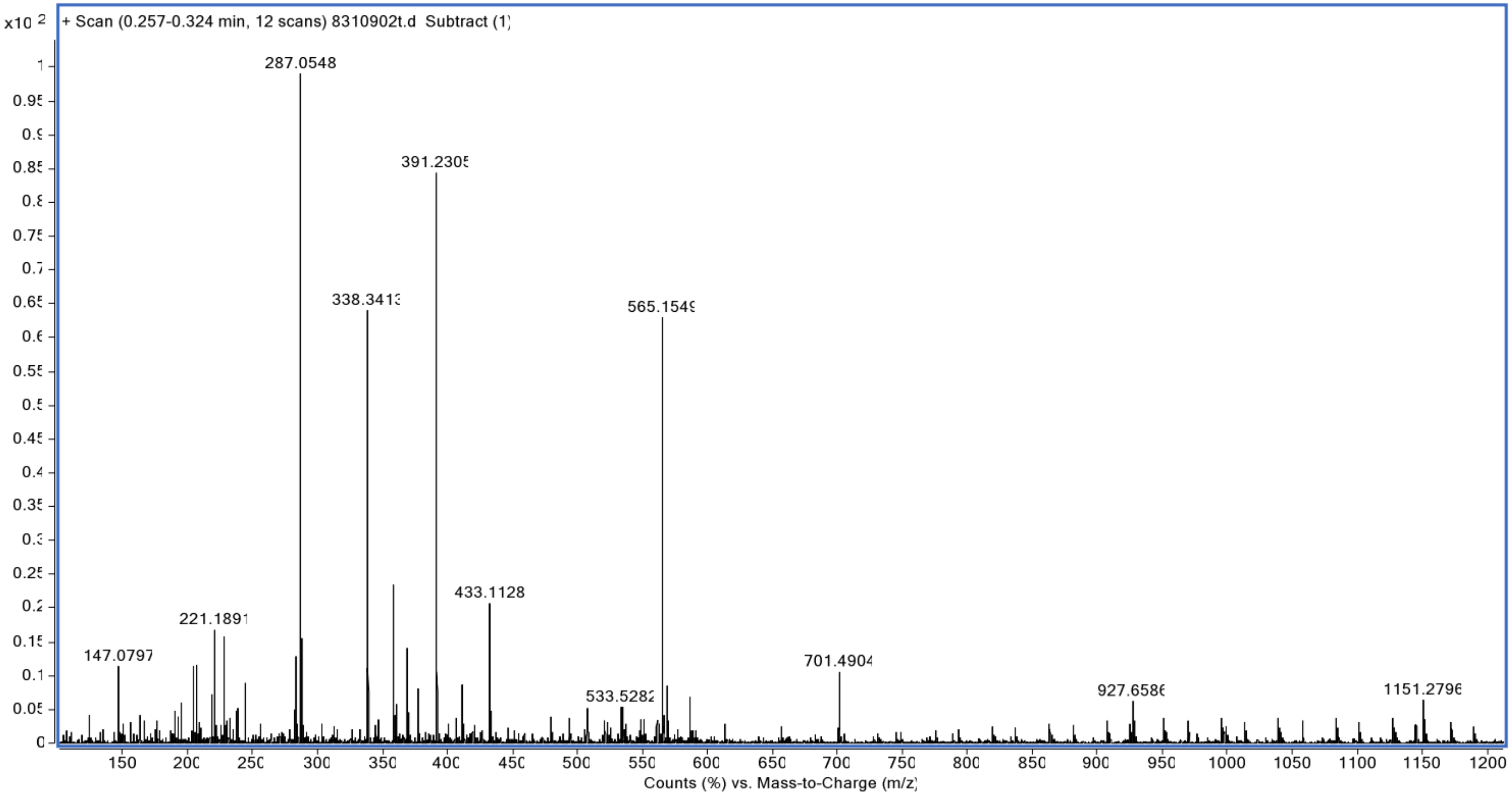

Figure S9 ESI-MS spectrum of compound 3
